# Fractional Exhaled Nitric Oxide (FeNO) in Patients with Stable Chronic Obstructive Pulmonary Disease: Short-Term Variability and Potential Clinical Implications

**DOI:** 10.3390/jpm12111906

**Published:** 2022-11-16

**Authors:** Pasquale Ambrosino, Salvatore Fuschillo, Mariasofia Accardo, Marco Mosella, Antonio Molino, Giorgio Alfredo Spedicato, Andrea Motta, Mauro Maniscalco

**Affiliations:** 1 Istituti Clinici Scientifici Maugeri IRCCS, Cardiac Rehabilitation Unit of Telese Terme Institute, 82037 Telese Terme, Italy; 2 Istituti Clinici Scientifici Maugeri IRCCS, Pulmonary Rehabilitation Unit of Telese Terme Institute, 82037 Telese Terme, Italy; 3 Department of Clinical Medicine and Surgery, Federico II University, 80131 Naples, Italy; 4 Department of Statistics and Quantitative Methods, Milano-Bicocca University, 20126 Milan, Italy; 5 Institute of Biomolecular Chemistry, National Research Council, 80078 Pozzuoli, Italy

**Keywords:** chronic obstructive pulmonary disease, biomarker, nitric oxide, chronic disease, disability, exercise, rehabilitation, outcome

## Abstract

Background: The use of fractional exhaled nitric oxide (FeNO) has been proposed for identifying and monitoring eosinophilic airway inflammation in chronic obstructive pulmonary disease (COPD). To explore the clinical utility of FeNO in COPD, we aimed to assess its short-term variability in a clinically stable COPD cohort. Methods: Consecutive COPD patients, formerly smokers, underwent FeNO assessment at the baseline and six time-points through serial sampling spaced 3 days apart. Results: A total of 41 patients (mean age 72.9, 87.8% males) showed a median baseline value of FeNO of 11.7 (8.0–16.8) ppb. A weak linear relationship was documented between baseline FeNO values and both eosinophil counts (r = 0.341, *p* = 0.029) and the percentage of eosinophils (r = 0.331, *p* = 0.034), confirmed in multiple linear regressions after adjusting for steroid use. The overall individual variability of FeNO between time-points was 3.90 (2.53–7.29) ppb, with no significant difference in the distribution of FeNO values measured at different time-points (*p* = 0.204). A total of 28 (68.3%) patients exhibited FeNO always below the 25 ppb cut-off at all determinations, while the remining 13 (31.7%) had at least one value above the established limit. Interestingly, none of these 13 participants had FeNO stably above 25 ppb, all showing at least one normal value during serial sampling. Compared to these patients with more fluctuating values, the 28 with stably normal FeNO only exhibited a significantly higher body weight (80.0 ± 18.2 kg vs. 69.0 ± 8.8 kg, *p* = 0.013) and body mass index (29.7 ± 6.5 kg/m^2^ vs. 25.9 ± 3.7 kg/m^2^, *p* = 0.026), confirmed in multiple logistic regressions after adjusting for major potential confounders. Conclusions: A certain degree of FeNO variability, apparently unrelated to eosinophil counts but somehow influenced by body weight, must be considered in COPD patients. Further studies are needed to clarify whether this biomarker may be effectively used to plan more personalized pharmacological and rehabilitation strategies in this clinical setting.

## 1. Introduction

With a worldwide prevalence of 10.3% among people aged 30–79 years [1], chronic obstructive pulmonary disease (COPD) is a chronic inflammatory airway disease characterized by irreversible and progressive airflow limitation, with parenchyma destruction and subsequent remodeling [2]. Over the past few decades, COPD has emerged as a growing public health problem, given the enormous burden in terms of comorbidities [3,4], disability [5,6,7], social costs [8,9,10], and rehabilitation needs [11,12,13].

Although the pathophysiological mechanisms of COPD have been extensively investigated [14], many aspects remain to be clarified given the heterogeneous clinical course in terms of severity and progression of the disease [15]. Airway inflammation in COPD is known to be mainly driven by a type 1 immune response [14]. However, type 2 inflammation also appears to play a significant role in both the stable state and during exacerbations [16], with up to 40% of COPD patients presenting some degree of airway eosinophilic inflammation [17,18].

Fractional exhaled nitric oxide (FeNO) is considered a reliable surrogate marker for type 2 inflammation of the airways and has acquired an important role in asthma, supporting diagnosis and helping to monitor response and adherence to anti-inflammatory treatment [19]. In contrast, given the pathophysiological complexity of COPD and the effect of cigarette smoking on FeNO [20], its potential clinical utility in this clinical setting is still debated [21]. Recently, it has been hypothesized that FeNO might reflect the presence of airway eosinophilia also in COPD patients [22], with increased FeNO levels being related to a more severe phenotype and frequent exacerbations [23,24]. Therefore, the use of FeNO, alone or in combination with other biomarkers (e.g., blood eosinophil counts), has been proposed for identifying COPD patients who may benefit from specific therapies, including monoclonal antibodies, while monitoring efficacy or adherence to treatments [22,25].

However, data on the variability and reproducibility of FeNO in patients with COPD are still scarce and heterogenous [26,27]. Similarly, the clinical and demographic variables affecting the stability of this parameter in this patient population are largely unknown [28,29]. Therefore, guiding some relevant therapeutic choices based on a single FeNO determination may lead to inappropriate prescriptions in COPD, thus pointing out the need for an investigation of FeNO variability in this clinical setting.

To address this issue, we aimed to assess the short-term variability of FeNO in a clinically stable COPD cohort through serial sampling spaced 3 days apart.

## 2. Materials and Methods

### 2.1. Patients

From April 2020 to November 2020, consecutive COPD patients referred to the Respiratory Unit of Istituti Clinici Scientifici Maugeri IRCCS, Telese Terme, Italy were evaluated for entry into this pilot study. To be included in the protocol, patients had to meet the following criteria: diagnosis of COPD according to the American College of Physicians, the American College of Chest Physicians, the American Thoracic Society (ATS), and European Respiratory Society (ERS) guidelines [30]; smoking cessation for at least 6 months; no exacerbation in the past 4 weeks. Exclusion criteria were: a history of allergies, lung surgery or drug/alcohol abuse; any upper or lower airway disease other than COPD; any chronic condition of rheumatological, infectious or gastrointestinal etiology; any condition that could affect the patient’s ability to undergo FeNO collection or to perform study protocol activities. Whenever applicable, the study followed the Strengthening the Reporting of Observational Studies in Epidemiology (STROBE) reporting guidelines [31]. The protocol was conducted according to the principles of the Helsinki Declaration after being approved by the local Ethics Committee (ref. n. ICS-03/20).

### 2.2. Study Procedures

Following signature of the informed consent by each study participant, the main clinical and demographic data were gathered. Moreover, all patients underwent spirometry (Vmax^®^ Encore, Vyasis Healthcare, Milan, Italy) and blood gas analysis in ambient air (ABL 825^®^ FLEX BGA, Radiometer Medical Aps, Copenhagen, Denmark) according to ATS/ERS guidelines [32,33,34], to quantify post-bronchodilator forced expiratory volume in 1 s (FEV_1_), forced vital capacity (FVC), FEV_1_/FVC ratio, and arterial blood gas [oxygen (PaO_2_) and carbon dioxide tension (PaCO_2_)]. A venous blood sample was also analyzed in all study participants to measure white blood cell counts. Exercise performance was tested by evaluating the six-minute walking distance (6MWD) [35].

A trained operator measured FeNO in parts per billion (ppb) using an automated electrochemical instrument (HypAir FeNO^®^, Médisoft, Sorinnes, Belgium) following a standardized procedure which has been detailed elsewhere [36]. In brief, participants were studied in the sitting position, starting from total lung capacity and exhaling against a positive pressure to generate an exhalation flow rate of 50 mL/s, kept constant by means of a visual biofeedback on the display of the device. With high measurement consistency expressed by a linearity error of <0.5% reported by the manufacturer, HypAir FeNO^®^ exhibited high precision and overall good compliance. Whenever applicable, FeNO was measured in all study participants at the baseline and at each time-point through serial sampling spaced 3 days apart (i.e., after 3, 6, 9, 12, 15, and 18 days). At the baseline, FeNO was assessed before post-bronchodilator functional spirometry. At different time-points, a single measurement was taken for each participant. If the first measurement failed, the entire procedure was repeated. A FeNO value of less than 25 ppb was considered normal/low [37].

### 2.3. Statistical Analyses

Statistical analyses were carried out with an IBM SPSS Statistics 22.0 system (Chicago, IL, USA). Continuous data were expressed as mean ± standard deviation or median (1st–3rd quartile) in case of a skewed distribution, while categorical variables were summarized as relative frequencies. The overall individual variability of FeNO over time was expressed as the standard deviation of measurements. We used Pearson or Spearman correlation coefficients to examine the relationship between continuous variables. When comparing continuous variables, given the unequal distribution of sample sizes, the independent t-test of Welch was used. We resorted to the Mann–Whitney U-test to compare distributions in case of values with a skewed non-Gaussian distribution. We used the Friedman test to compare the distribution of FeNO values at different time-points. Moreover, a repeated-measures analysis of variance (ANOVA) with mixed-effects modelling was performed to evaluate the interaction of categorical variables with time and associated changes in FeNO over time. The Pearson’s chi-squared test with Yates continuity correction was used to compare dichotomous variables. Linear and binary logistic regression analyses were used to adjust for potential confounders and identify predictors. A *p*-value <0.05 (2-sided) was considered statistically significant. An *a priori* power analysis was not conducted for this exploratory pilot study.

## 3. Results

A total of 5 (9.6%) out of the 52 eligible patients were not considered due to poor compliance in the procedure for FeNO measurement, while 6 (11.5%) dropped out before completion of the project requirements (Supplemental Figure S1). Thus, 41 Caucasian patients (mean age 72.9, 87.8% males) were included in the final analysis (Table 1).

Our study population consisted of stable elderly COPD patients, having a predicted mean FEV_1_ of 49.0 ± 14.4% and an FEV_1_/FVC ratio of 45.9 ± 12.5. According to the Global Initiative for Chronic Obstructive Lung Disease (GOLD) classification [38], most participants (78.1%) were in Group D (high risk of exacerbation, more symptoms), while the remaining 21.9% were in Group C (high risk of exacerbation, less symptoms). Patients were generally overweight, with a median body mass index (BMI) of 28.1 kg/m^2^, being generally self-sufficient and able to walk alone or with aids for a mean 6MWD of 175.8 m. All study participants were former smokers, and, at the time of enrollment, all were under treatment with inhaled corticosteroids (ICS), long-acting β_2_-agonists (LABA), and/or long-acting muscarinic antagonists (LAMA).

The median baseline value of FeNO in the study population was 11.7 (8.0–16.8) ppb. A weak positive linear relationship was documented between baseline FeNO values and eosinophil counts (r = 0.341, *p* = 0.029) and, similarly, between baseline FeNO and the percentage of eosinophils (r = 0.331, *p* = 0.034). In multiple linear regressions, after adjusting for steroid use, both eosinophil counts (β = 0.311, *p* = 0.048) and the percentage of eosinophils (β = 0.313, *p* = 0.044) were found to be independent predictors of FeNO.

**Figure 1:**
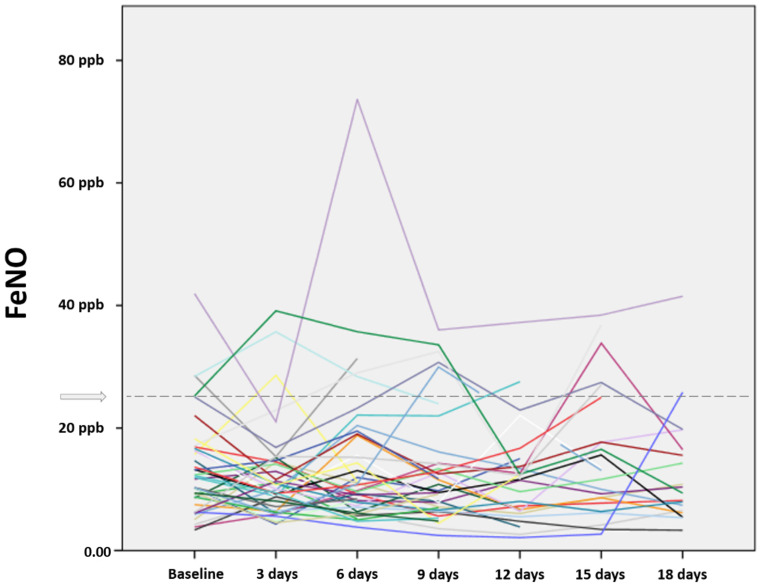
Individual fractional exhaled nitric oxide (FeNO) values in 41 stable chronic obstructive pulmonary disease (COPD) patients measured at different time-points spaced 3 days apart. Different colors indicate different patients. The dashed grey line indicates the 25 ppb cut-off.

Ranging from a minimum value of 6.10 (4.79–9.35) ppb to a maximum value of 16.9 (11.3–28.1) ppb, the overall individual variability of FeNO between time-points was 3.90 (2.53–7.29) ppb. No significant difference was found in the distribution of FeNO values measured at different time-points (*p* = 0.204). Overall, out of 41 COPD patients, a total of 28 (68.3%) remained with stable FeNO values below the 25 ppb cut-off at all time-points, while the remining 13 (31.7%) patients had at least one determination above the established limit. Interestingly, none of these 13 participants remained with stable FeNO values above 25 ppb at all time-points, showing at least one normal value during serial sampling spaced 3 days apart (Figure 1). The mixed-effects ANOVA showed no significant interaction of the two groups with time and associated changes in FeNO over time (*p* = 0.310).

As shown in Table 1, no difference was found between the two groups in the main demographic and clinical variables, related to spirometry parameters, GOLD stage, arterial blood gas, blood cell counts, including eosinophils (*p* = 0.570), and treatments, including inhaled corticosteroids (*p* = 0.693) and their dose (*p* = 0.718). However, patients who remained with stable FeNO values below the cut-off of 25 ppb exhibited a significantly higher body weight (80.0 ± 18.2 kg vs. 69.0 ± 8.8 kg, *p* = 0.013) as compared to those with at least one determination above the normal limit. This result was substantially confirmed in a multivariate analysis, after adjusting for age, sex, height, pulmonary function, eosinophil count, and steroid use (β = 0.938, *p* = 0.042). Accordingly, a significantly higher BMI (29.7 ± 6.5 kg/m^2^ vs. 25.9 ± 3.7 kg/m^2^, *p* = 0.026) was also reported in patients with normal FeNO values at all time-points, confirmed after adjusting for potential confounders (β = 0.845, *p* = 0.046).

## 4. Discussion

This pilot study is the first attempt to explore short-term FeNO variability in a cohort of stable COPD patients. Confirming that FeNO is a biomarker of eosinophilic airway inflammation, our findings support its potential utility in monitoring and managing COPD. However, a certain degree of instability in its values, apparently unrelated to eosinophil counts but somehow influenced by body weight, has emerged in a non-negligible percentage of COPD patients, thus suggesting that a repeated sampling rather than a single determination may be required to increase the diagnostic and prognostic value of FeNO in this clinical setting.

While induced sputum has long been considered the “gold standard” for eosinophilic airway inflammation [39], a good level of association between FeNO, eosinophils in sputum, and eosinophils in blood has been documented [40]. Therefore, FeNO has been widely adopted in routine clinical practice during recent years as a surrogate marker of airway eosinophilia, due to its non-invasiveness and ease of execution [41]. In particular, FeNO has proven its clinical utility as a biomarker of T helper 2 (Th2) airway inflammation in asthma, supporting diagnosis and helping to monitor response and adherence to steroids [19]. In COPD patients, eosinophilia has been associated with exacerbations and hyperinflation [42], thus leading the GOLD guidelines to recommend the use of ICS in patients with eosinophilic phenotype [43,44]. Overall, although there is currently no general consensus on this issue [45], there is no reason to overlook the potential clinical utility of FeNO for identifying and monitoring treatment efficacy in the subgroup of COPD patients with airway eosinophilia.

The mechanisms underlying eosinophilic inflammation in up to 40% of COPD patients [17,18], both during exacerbations and in stable periods [46,47], have not been fully elucidated but they do not seem to be identical to asthma [48]. In particular, while allergen-specific Th2 cells drive the pathogenesis of allergic asthma, non-specific innate lymphoid cells (ILC2s) seem to be involved in the pathogenesis of eosinophilic inflammation in COPD as well as non-allergic asthma [49,50]. ILC2s have been discovered in relatively recent times, potentially linking the innate and adaptive immune responses in chronic diseases involving type 2 inflammation [51]. It has been proposed that ILC2s in COPD patients may be activated by inflammatory mediators released by the pulmonary epithelium and other structural cells [52], thus resulting in the synthesis of a number of cytokines, including interleukin (IL)-4, IL-5 and IL-13, which are, in turn, known to participate in airway eosinophilia and parenchyma remodeling [53].

It has been demonstrated that COPD patients with such eosinophilic inflammation tend to experience uncontrolled symptoms despite optimal pharmaceutical treatment [48]. With the aim of more targeted therapy, this has led to testing some new anti-eosinophilic molecules in COPD, such as monoclonal antibodies [54], which have already shown efficacy in asthma [55,56]. Thus, if eosinophilic inflammation may represent a new therapeutic target in COPD, blocking the IL-5 signaling pathway by directly binding the alpha chain of the cytokine (e.g., mepolizumab, reslizumab) or inhibiting its receptor (*e.g.*, benralizumab) may represent one the most effective strategies [48]. Promising but sometimes contradictory results in terms of eosinophil depletion, exacerbation rate, and lung function have so far been obtained from randomized clinical trials (RCTs) and cohort studies on mepolizumab [57,58,59]. On the other hand, more disappointing results have been obtained in RCTs on benralizumb [60], while clinical trials on the use of the anti-IL-4Rα dupilumab in patients with moderate-to-severe COPD and type 2 inflammation are still ongoing (NCT03930732). Overall, it seems clear that the use of these and other biologic medications targeting the eosinophilic phenotype remains a promising pharmacological approach but needs further investigations, most of which are underway. In the meantime, the identification of novel biomarkers for predicting response is mandatory [61].

Our findings are in line with the growing body of evidence suggesting a certain degree of correlation between FeNO and the eosinophilic phenotype [40]. Thus, if FeNO is a surrogate marker for eosinophilic airway inflammation [62], it can be argued that it may also be a good candidate biomarker in COPD, helping to monitor response to corticosteroids or monoclonal antibodies [63]. In a previous meta-analysis, a decrease in FeNO among COPD patients treated with inhaled corticosteroids was documented, particularly in former smokers [64]. However, ours is the first study to report on short-term FeNO variability, suggesting that a single determination in COPD patients may not be sufficient to identify those who may benefit from specific therapies while monitoring efficacy or adherence to treatments, as is routine in asthma [65,66]. The main objective of our investigation was not to clarify the relationship between FeNO and the eosinophilic phenotype in COPD patients, which was, incidentally, found to be weak and has already been reported in other studies with sometimes conflicting findings [67]. Our aim was rather to explore the stability of FeNO in this clinical setting, identifying the potential clinical or demographic variables related to its variability. Of interest, the only clinical variable potentially impacting FeNO stability over time was found to be body weight, even after adjusting for major potential confounders, including steroid use. Although conflicting results have been reported in the literature on the relationship between FeNO and body weight [68], the latter finding may (at least in part) be in line with our previous studies documenting low alveolar and bronchial nitric oxide in uncomplicated obesity [69] and its restoration after weight loss [70]. A mechanical rather than biological explanation was provided in these previous studies [69,70], calling into question the reduced diffusing capacity and the consistently increased airway resistance of obese subjects. On the other hand, it was also speculated that the predominantly neutrophilic inflammation in the case of weight excess may somehow hide an airway eosinophilic pattern. Although preliminary, these findings offer an interesting insight in the relationship between body weight and the eosinophilic phenotype in COPD, suggesting the need for further clinical and laboratory investigations.

Some potential limitations of this study should be addressed. A major limitation of our protocol is the relatively small sample. Therefore, the above findings need confirmation in larger studies with a robust design, better considering the high heterogeneity of COPD in terms of severity and disease progression. In this regard, it should be noted that, based on the admission criteria of COPD patients to our Respiratory Unit, only participants of Group C and D were considered in our protocol. Therefore, our findings are not generalizable to less severe forms of the disease. Another potential limitation of this study is the inclusion of only Caucasian patients, all coming from the Campania Region in Italy. As significant racial differences in the comorbidity profile of COPD patients have been documented [71], our results on short-term FeNO variability cannot be generalized to all ethnicities. Similarly, given the predominance of males in our study population, sex-biased information has also been provided. The epidemiological data [1] of a disproportionally higher prevalence of COPD among males may partially compensate for the above limitation. A further weakness of our study is that the results are limited to former smokers. However, the decision to specifically exclude current smokers from our protocol was due to the potential interference of cigarette smoke on FeNO values [28]. Finally, it should be noted that the 3-day interval between each determination was arbitrary, conditioned by purely technical and organizational reasons in our Respiratory Unit. Although a daily FeNO assessment would have been optimal, we are confident that 3 days (the shortest possible time interval in our protocol) would be sufficient to explore short-term FeNO variability as part of a preliminary pilot research.

## 5. Conclusions

Our preliminary findings suggest that some degree of short-term variability must always be taken into account when using FeNO as a biomarker of airway eosinophilic inflammation in COPD patients, thus suggesting that a repeated sampling rather than a single determination may be required to increase its diagnostic and prognostic value in this clinical setting. These exploratory findings, although requiring confirmation, may have relevant repercussions in clinical practice and future research on the usefulness of this biomarker in COPD. Further robust investigations should clarify whether FeNO may be effectively used to identify patients who may benefit from specific therapies, including monoclonal antibodies, while monitoring efficacy and adherence to treatments.

## Figures and Tables

**Table 1:** Main demographic and clinical characteristics of chronic obstructive pulmonary disease (COPD) patients.

Variable	Overall	FeNO ≤ 25 ppb	FeNO > 25 ppb	*p*-Value
	41	28	13	
**Demographic**				
Age, years	72.9 ± 7.4	74.2 ± 6.7	70.1 ± 8.2	0.098
Males, n (%)	36 (87.8)	24 (85.7)	12 (92.3)	0.930
**Anthropometric**				
Height, cm	163.6 ± 5.5	163.6 ± 5.7	163.5 ± 5.3	0.955
Weight, kg	76.5 ± 16.6	80.0 ± 18.2	69.0 ± 8.8	**0.013**
BMI, kg/m^2^	28.1 (24.1–32.2)	29.7 ± 6.5	25.9 ± 3.7	**0.026**
**Pulmonary function tests (PFTs)**				
FEV_1_, L	1.17 ± 0.38	1.19 ± 0.33	1.13 ± 0.49	0.740
FEV_1_, % predicted	49.0 ± 14.4	51.1 ± 14.4	44.3 ± 13.9	0.161
FVC, L	2.59 ± 0.66	2.55 ± 0.63	2.66 ± 0.72	0.651
FVC, % predicted	79.0 (70.0–95.5)	83.5 ±16.9	81.7 ± 14.3	0.731
FEV_1_/FVC	45.9 ±12.5	47.7 ± 12.5	41.9 ± 11.9	0.169
**GOLD stage**				
Group C, n (%)	9 (21.9)	7 (25.0)	2 (15.4)	0.774
Group D, n (%)	32 (78.1)	28 (75.0)	11 (84.6)	0.774
**Blood gas analysis**				
SpO_2_, %	94.0 (91.8–95.0)	93.5 (91.8–95.0)	94.0 (90.1–95.0)	0.631
PaO_2_, mmHg	60.1 ± 9.1	59.6 ± 8.4	60.9 ± 10.7	0.775
PaCO_2_, mmHg	42.7 ± 4.1	42.7 ± 4.4	42.9 ± 4.0	0.924
pH	7.42 ± 0.04	7.42 ± 0.04	7.42 ± 0.03	0.887
**Laboratory parameters**				
Leucocytes, 10^3^/µL	7.90 (6.35–9.40)	8.20 (6.80–9.40)	7.20 (5.75–9.95)	0.468
Neutrophils, 10^3^/µL	4.95 ± 1.92	4.99 ± 1.78	4.87 ± 2.28	0.859
Neutrophils, %	61.0 ± 12.5	61.0 ± 12.8	61.1 ± 12.2	0.985
Basophils, 10^3^/µL	0.05 (0.01–0.08)	0.05 (0.01–0.08)	0.05 (0.01–0.06)	0.497
Basophils, %	0 (0–1.00)	0 (0–1.00)	0 (0–1.00)	0.879
Monocytes, 10^3^/µL	0.68 (0.59–0.83)	0.69 (0.59–0.83)	0.66 (0.60–0.85)	1.000
Monocytes, %	9.17 ± 2.93	8.86 ± 3.03	9.85 ± 2.70	0.304
Eosinophils, 10^3^/µL	0.19 (0.09–0.32)	0.18 (0.09–0.29)	0.19 (0.11–0.40)	0.570
Eosinophils, %	3.00 (1.00–3.50)	3.00 (1.00–3.00)	3.00 (1.50–4.50)	0.497
Lymphocytes, 10^3^/µL	1.79 (1.49–2.34)	1.90 (1.51–2.43)	1.70 (1.31–2.03)	0.216
Lymphocytes, %	26.0 (18.0–33.5)	25.0 (18.3–33.8)	26.0 (14.5–34.0)	0.446
CRP, mg/dL	9.8 (5.6–17.4)	9.8 (5.6–15.6)	11.8 (5.5–20.3)	0.642
**Pharmacological therapy**				
ICS, n (%)	25 (61.0)	16 (57.1)	9 (69.2)	0.693
FP equivalent dose, µg/day	1000 (500–1000)	1000 (500–1000)	1000 (750–1000)	0.718
LABA, n (%)	31 (75.6)	22 (78.6)	9 (69.2)	0.797
LAMA, n (%)	31 (75.6)	21 (75.0)	10 (76.9)	1.000
**Exercise capacity**				
6MWD, meters	175.8 ± 43.7	171.0 ± 47.4	185.8 ± 34.3	0.272

BMI: body mass index; FEV_1_: forced expiratory volume in 1 s; FVC: forced vital capacity; GOLD: Global Initiative for Chronic Obstructive Lung Disease; SpO_2_: pulse oxygen saturation; PaO_2_: arterial oxygen tension; PaCO_2_: arterial carbon dioxide tension; CRP: C-reactive protein; ICS: inhaled corticosteroids; FP: fluticasone propionate; LABA: long-acting β_2_-agonists; LAMA: long-acting muscarinic antagonists; 6MWD: 6-min walk distance. Continuous data are presented as mean ± standard deviation or median (1st–3rd quartile) in case of skewed distribution. Categorical variables are summarized as relative frequencies. A *p*-value of <0.05 was considered statistically significant (bold font).

## Data Availability

The data supporting the findings of this study are available from the corresponding authors upon reasonable request.

## Supplementary Materials

The following supporting information can be downloaded at: https://www.mdpi.com/article/10.3390/jpm12111906/s1, Supplemental Figure S1: Flow chart of study participants.
