# Supplementary material for: Fractional Exhaled Nitric Oxide (FeNO) in Patients with Stable Chronic Obstructive Pulmonary Disease: Short-Term Variability and Potential Clinical Implications
Source: J Pers Med. 2022 Nov 16;12(11):1906. doi: 10.3390/jpm12111906 (PMC9699194; doi:10.3390/jpm12111906)
Supplement: Supplementary file 1 [file jpm-12-01906-s001.zip › jpm-1980015-supplementary.pdf]

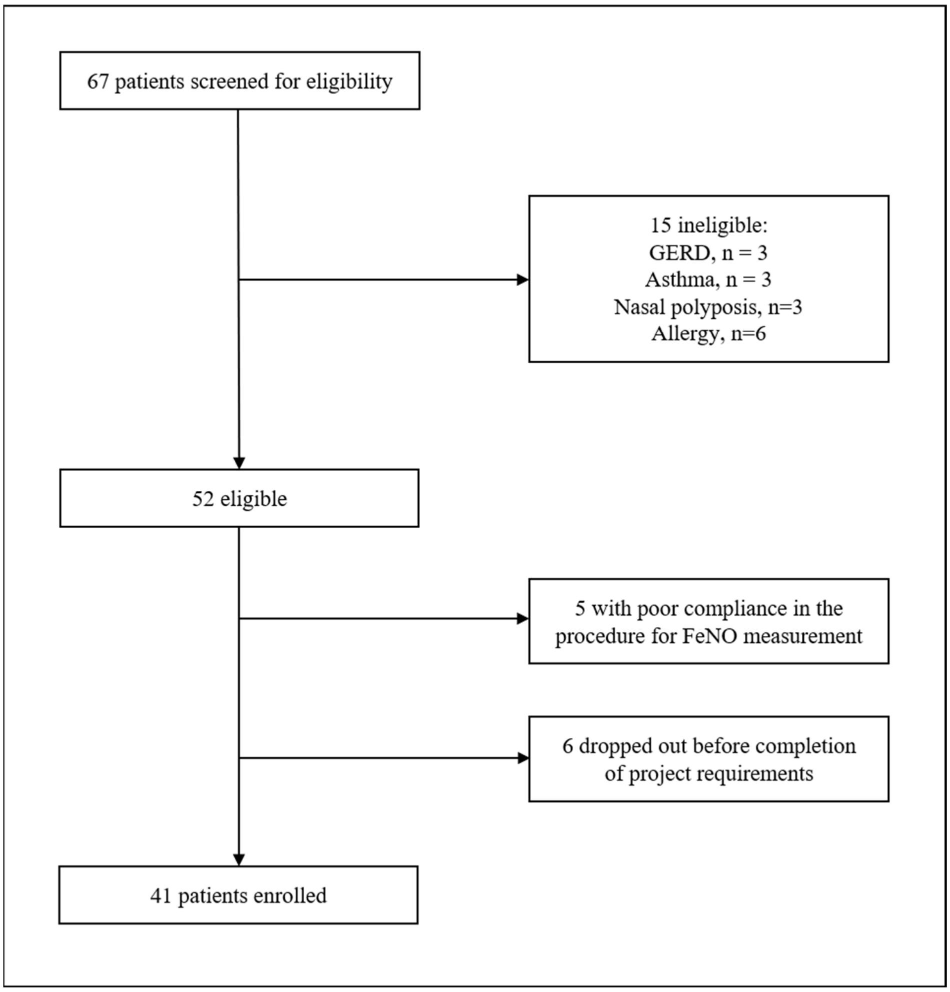

Supplemental Figure S1. Flow chart of study participants. GERD: gastroesophageal reflux disease.
